# MicroRNA-24 Modulates Aflatoxin B1-Related Hepatocellular Carcinoma Prognosis and Tumorigenesis

**DOI:** 10.1155/2014/482926

**Published:** 2014-04-08

**Authors:** Yi-Xiao Liu, Xi-Dai Long, Zhi-Feng Xi, Yun Ma, Xiao-Ying Huang, Jin-Guang Yao, Chao Wang, Tian-Yu Xing, Qiang Xia

**Affiliations:** ^1^Department of Liver Surgery, Ren Ji Hospital, School of Medicine, Shanghai Jiao Tong University, Dongfang Road, No. 1630, Shanghai 200127, China; ^2^Department of Pathology, Youjiang Medical College for Nationalities, Baise 533000, China; ^3^Department of Pathology, The Affiliated Tumor Hospital, Guangxi Medical University, Nanning 530021, China; ^4^Department of Medicine, The Affiliated Hospital of Youjiang Medical College for Nationalities, Baise 533000, China

## Abstract

MicroRNA-24 (miR-24) may be involved in neoplastic process; however, the role of this microRNA in the hepatocellular carcinoma (HCC) related to aflatoxin B1 (AFB1) has not been well elaborated. Here, we tested miR-24 expression in 207 pathology-diagnosed HCC cases from high AFB1 exposure areas and HCC cells. We found that miR-24 was upregulated in HCC tumor tissues relative to adjacent noncancerous tissue samples, and that the high expression of miR-24 was significantly correlated with larger tumor size, higher microvessel density, and tumor dedifferentiation. Additionally, this microRNA overexpression modified the recurrence-free survival (relative hazard ratio [HR], 4.75; 95% confidence interval [CI], 2.66–8.47) and overall survival (HR = 3.58, 95% CI = 2.34–5.46) of HCC patients. Furthermore, we observed some evidence of joint effects between miR-24 and AFB1 exposure on HCC prognosis. Functionally, miR-24 overexpression progressed tumor cells proliferation, inhibited cell apoptosis, and developed the formation of AFB1-DNA adducts. These results indicate for the first time that miR-24 may modify AFB1-related HCC prognosis and tumorigenesis.

## 1. Introduction

Hepatocellular carcinoma (HCC) remains a life-threating malignancy, accounting for more than 90% of primary liver cancer. This tumor is the sixth most commonly occurring cancer worldwide, with an estimated 600,000 new cases per year. More than 80% of all HCC cases occurred in developing countries, and approximately 55% of all cases occur in China (especially in the southeast areas such as Guangxi) [1, 2]. Because of the very poor prognosis resulting from metastasis and reoccurrence, HCC is the third leading cause of cancer-related deaths in the world [3]. Therefore, improvement in the therapy of recurrent or metastatic HCC now depends on improving our understanding of the complex molecular mechanisms governing the progression and aggressiveness of the disease, and looking for valuable prognostic markers and therapeutic targets.

MicroRNAs are small, endogenous, and noncoding RNAs that regulate the translation of protein-coding genes by repressing translation of protein-coding mRNA or enhancing mRNA degradation [4, 5]. Deregulation of microRNAs has been reported to modulate normal cell growth and differentiation, potentially leading to a variety of disorders, including cancer [6, 7]. To date, there are more than 2000 annotated human mature microRNAs in the official registry (the MicroRNA Registry) [8]. Among these microRNAs, miR-24 has been shown to regulate the carcinogenesis of a variety of cancers including HCC [9–13]. However, association between this microRNA and aflatoxin B1 (AFB1)-related HCC prognosis has not yet been elucidated. Here, we evaluated whether miR-24 expression modified HCC tumorigenesis and prognosis.

## 2. Materials and Methods

### 2.1. HCC Patients

During the period from January 2004 to December 2008, all incident cases with the I-II tumor-nodes-metastasis (TNM) stage HCC were recruited at the affiliated hospitals Guangxi Medical University and Youjiang Medical College for Nationalities. All patients were the residents of Guangxi Zhuang Autonomous Region, a high AFB1 exposure area. A total of 207 HCC cases, including 138 patients previously studied [14–16], were included for the present study. All cases were histopathologically confirmed and previously untreated with chemotherapy or radiotherapy. In this study, the response rate for the cases has been about 95%. The study protocol was been carried out in accordance with “Ethical Principles for Medical Research Involving Human Subjects” (World Medical Association Declaration Of Helsinki, 2004) and approved by institutional review boards from Guangxi Cancer Institute and the Medical Research Council from the corresponding hospitals.

### 2.2. Samples and Data Collection

After informed consent was obtained, surgically removed tumor samples and adjacent noncancerous tissue samples (at least 5 cm from the margin of the tumor) of all cases were collected for analyzing miR-24 expression levels and AFB1-DNA adduct levels. Demographic information (including sex, age, ethnicity, hepatitis B virus [HBV], and hepatitis C virus [HCV] infection) and clinical pathological data (including cirrhosis, tumor size, tumor differentiation, and tumor stage) were collected in the hospitals using a standard interviewer-administered questionnaire and/or medical records by a Youjiang Cancer Institution staff member. In this study, those with hepatitis B surface antigen (HBsAg) positive and anti-HCV positive in their peripheral serum were defined as groups infected with HBV and HCV. Liver cirrhosis was diagnosed by pathological examination, and stages of tumor were confirmed according to the tumor nodes metastasis (TNM) staging system. For tumor grading, Edmondson and Steiner grading system was used to evaluate the differentiation status of HCC in this study [17].

For survival analysis, we followed all HCC cases and more detailed follow-up information was described in our previous studies [14–16, 18]. Briefly, all patients underwent serial monitoring every 2 months for the first 2 years and semiannually thereafter for detection of any recurrence. In the present study, the last follow-up day was set on August 31, 2013, and survival status was confirmed by means of clinic records and patient or family contact. The duration of the duration of overall survival (OS) was defined as from the date of curative treatment to the date of death or last known date alive; whereas the recurrence-free survival (RFS) was defined as from the date of curative treatment to the date of tumor recurrence or last known date alive [14, 15].

### 2.3. DNA and RNA Detraction

Genomic DNA was extracted from HCC tumor tissues and SMMC-7721 cells in a 1.5 mL microcentrifuge tube for deparaffinization and proteinase K digestion, as described by standard procedures (Protocol #BS474, Bio Basic, Inc., Ontario, Canada). For RNA detraction, total RNA was isolated from tissue or cell cultures, using PureLink RNA minikit (cat number 12183018A, Ambion, USA) according to manufacturer's instructions.

### 2.4. AFB1 Exposure Analysis

In this study, AFB1 exposure levels were evaluated using the levels of AFB1-DNA adducts in tumor tissues. The amount of AFB1-DNA adducts in cancerous tissues samples was evaluated by means of competitive enzyme-linked immunosorbent assay (ELISA) [19]. To convert any N-7 adduct to AFB1-FAPy adducts, DNA was treated with 15 mM Na_2_CO_3_ and 30 mM NaHCO_3_ (pH 9.6) for 2 hours, precipitated with 2.5 volumes of 95% ethanol, and then redissolved in 10 mM Tris-HCl (pH 7.0). The DNA samples were reprecipitated, dissolved in 1 × PBS, and denatured by boiling for 5 min. After that, AFB1-FAPy adducts were quantitated by ELISA using monoclonal antibody 6A10 (Novus Biologicals LLC, catalog number NB600-443). For analysis, AFB1-DNA adduct levels were divided into two groups: low level (≤2.87 *μ*mol/mol DNA) and high level (>2.87 *μ*mol/mol DNA), according to the average value of AFB1-DNA adduct levels among cases.

### 2.5. The Microvessel Density (MVD) Evaluation

In the present study, the angiogenesis of cancerous tissues was assessed using the IHC staining of CD31 (cat number 2011101101, Gene Tech (Shanghai) Company Limited, Shanghai, China). At ×200 magnification, vessel counts were made of all distinct brown staining endothelial cells in the cancerous regions over five fields in each slide. The microvessel density (MVD) was defined as the average value of the three readings. To analysis the relationship between miR-24 expression and angiogenesis in the cancerous tissues, the angiogenesis status was divided into two groups: low (≤50/×200 magnifications) and high (>50/×200 magnifications), according to the mean MVD of cancerous-tissues vessels.

### 2.6. MiR-24 Expression Assay

The level of miR-24 expression was analyzed using our previously published TaqMan quantitative reverse transcription-PCR technique [16]. In brief, RNA was reversed transcribed into cDNA using high capacity cDNA reverse transcription kit (cat number 4368814, Invitrogen) and TaqMan microRNA reverse transcription kit (cat number 4366596, Applied Biosystems [ABI], Carlsbad, CA), according to the manufacturer's instructions. In this study, U6 expression was used as an internal control. Real-time quantitative PCR with TaqMan probes (cat number 4427975, ABI) was performed in total volume of 5 *μ*L containing 1 × TaqMAN Universal Master Mix II (cat number 4440041, ABI), 1 × TaqMan microRNA probe and primer Mix, and about 15 ng of cDNA using the running conditions: 95°C for 30 s, and 50 cycles of 95°C for 15 s and 60°C for 1 min. All reactions were conducted in triplicate and controls were performed with no template or no reverse transcription for each gene. The cycle number at which the reaction crossed an arbitrarily placed threshold (CT) was determined for each gene. For the relative expression of miR-24 in cancer cells, miR-24 expression was normalized to endogenous controls U6 by the comparative CT method (2^−ΔΔCt^ method). For tissue samples, the relative amount of miR-24 to U6 was calculated as 2^−ΔCt^ method, where ΔCt = (Ct_miR-24_ − Ct_U6_). To analyse, miR-24 expression levels were divided into two groups: low expression group, 2^−ΔCt^ ≤ 2.95; and high expression group, 2^−ΔCt^ > 2.95, according to the average value among HCC cases.

### 2.7. Cell Lines and Culture

The QSG7701 cells (a kind of peritumoral liver cells) and HCC cells (including SMMC7721, HepG2, and HCCLM3) were obtained from the Cell Bank of Shanghai Institute of Cell Biology of the Chinese Academy of Sciences and Shanghai Xinran Ltd. (Shanghai, China). Cells were cultured in DMEM medium (HyClone, Thermo Fisher Scientific (China) CO., Ltd., Shanghai, China) containing high glucose and L-glutamine supplemented with 10% fetal bovine serum at 37°C in an atmosphere of 5% CO_2_/100% humidity. All experimental analyses were done with cells in logarithmic growth. Cells were determined to be free of mycoplasma.

### 2.8. AFB1 Toxicity Analysis

Cells were transfected with an miR-24 mimic, its inhibitor, its mock, or null control (GenePharma, China) using Lipofectamine 2000 (cat number 11668-027, Invitrogen Grand Island, NY, USA) according to the manufacturer's instructions. According to the types of mimics transfected, cell lines were divided into four groups: control group (control, by null mimics), mock group (mock, by mock mimics); miR-24 group (miR-24, by mature miR-24 mimics), and inhibitor group (inhibitor, by inhibitor of mature miR-24). In this study, transfection efficacy was evaluated as the ratio of transfected cells detected by the LV200 system to total cells obtained from three different regions at random and was about 40%. AFB1 toxicity value was evaluated as our previously published methods [15]. Briefly, 48 hours after transfections, cells were treated with AFB1 (Sigma) at final concentrations of 24 nM for 1 day, and then the DNA was extracted for AFB1-DNA adduct analysis.

### 2.9. Cell Transfection and Cell Proliferation and Apoptosis Assay

The cell proliferation assay was measured using a cell counting kit (CCK-8) assay (cat number CK04, Dojindo Corp., Japan) according to the manufacturer's instructions. A total of 2500 cells were seeded each well in a 96-well plate. Ten microliters of CCK-8 solution was added into 100 *μ*L of culture media and incubated for 2 hours at 37°C. Finally, the absorbance of optical density (at 450 nm) was measured using UV spectrophotometer at 72 hours after transfection. The assay was performed three times in eight replicates. To analyse, relative proliferation value (RPV) of different groups to control group was calculated as OD250_XG_/OD250_Ref_, where OD250_XG_ represented OD250 value for cell proliferation analysis for different groups, and OD250_Ref_ represented OD250 value for cell proliferation analysis for control group.

In this study, cell apoptosis was analyzed by the flow cytometry technique using Annexin V, FITC apoptosis detection kit (cat number AD10-10, Dojindo Corp., Japan). Cells were seeded 6-wells and the transfections were performed when cells reached 70% confluent. 48 hours after transfection, cells were harvested, washed, and resuspended for cell apoptosis analysis. To analyse, relative apoptosis value (RAV) of different groups to control group was calculated using the following formula:

(1) $$\begin{array}{c} \text{RAV}=\frac{{\text{PPC}}_{\text{XG}}}{{\text{PPC}}_{\text{Ref}}}, \end{array}$$

where PPC_XG_ represented the percent of positive cells with apoptosis for different groups, and PPC_Ref_ represented the percent of apoptosis positive cells for control group.

### 2.10. Statistical Analysis

MiR-24 expression among different tissues and cells was compared by independent two-sample *t*-test and Mann-Whitney *U* test for two groups, or one-way ANOVA with Bonferroni corrections for three or more than three groups. Nonconditional logistic regression was used to evaluate odds ratios (ORs) and 95% confidence intervals (CIs) for the effects of miR-24 expression on the pathological features of HCC (including AFB1-DNA adducts, tumor size, tumor differentiation, and MVD). Kaplan-Meier survival analysis (with the log-rank test) was used to evaluate the association between miR-24 expression and HCC prognosis. Hazard ratios (HRs) and 95% CIs for miR-24 expression were calculated from multivariate Cox regression model. In this study, a *P* value of less than 0.05 was considered statistically significant. All analyses were performed with the statistical package for social science (SPSS) version 18 (SPSS Institute, Chicago, IL, USA).

## 3. Results

### 3.1. The Characteristics of HCC Patients

Table 1 showed the demographic and clinic-pathological data of the cases. The present study comprised 207 HCC patients with 189 (91.3%) males and 18 (8.7%) females. The mean age was 46.9 ± 11.5 years. Among these patients, more than 80% of cases were infected by HBV, and most of them had liver cirrhosis. One hundred percent of patients featured HCC with I-II TNM stage and received the same curative resection treatment, according to Chinese Manage Criteria of HCC. During the follow-up period of these patients, 72 faced tumor recurrence with 61.3% of the 5-year RFS rate, and 115 died with 47.5% of the five-year OS rate.

### 3.2. AFB1 Exposure Levels Related to Poor Prognosis of HCC Patients

**Figure 1:**
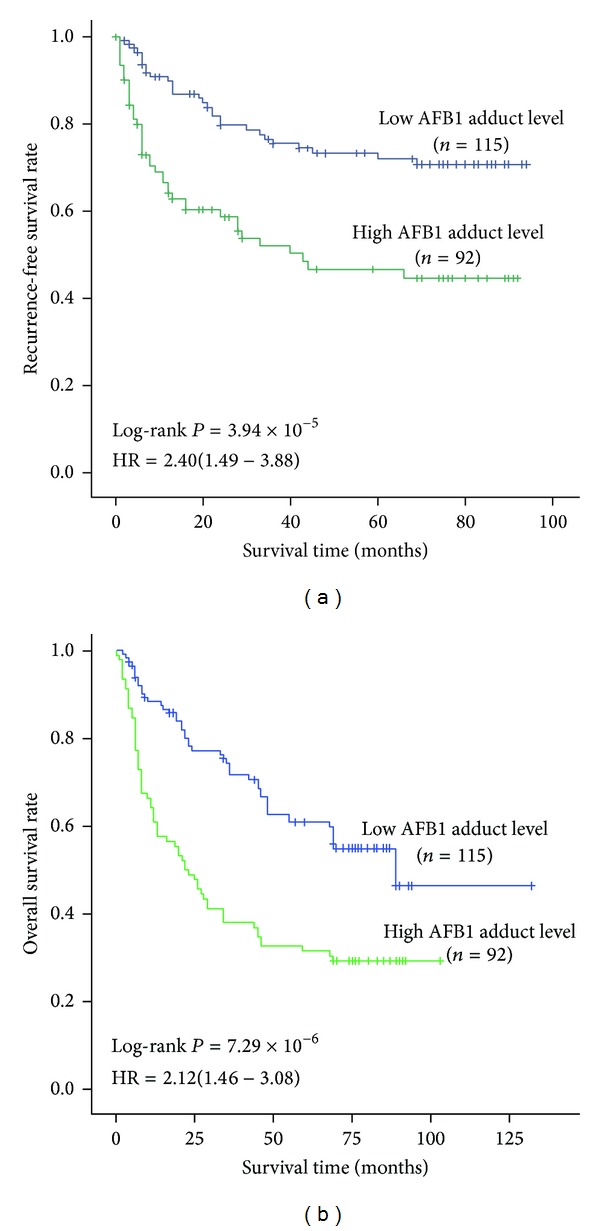
Association between survival and AFB1 exposure in 207 HCC cases receiving curative treatment. According to the average level of AFB1-DNA adducts in cancerous tissues, AFB1 exposure was divided into two groups: low exposure group (relative level ≤ 2.87 *μ*mol/mol DNA) and high exposure group (relative level > 2.87 *μ*mol/mol DNA). AFB1 exposure levels were associated with tumor reoccurrence-free survival (a) and overall survival (b) of HCC. Cumulative hazard function was plotted by Kaplan-Meier's methodology, and *P* value was calculated with two-sided log-rank tests. Relative hazard ratio (HR) and corresponding 95% CI of high AFB1 exposure (compared with low exposure) was calculated using multivariable cox regression model (including all significant variables).

In this study, we elucidated AFB1 exposure levels through testing AFB1-DNA adducts of DNA samples from cancerous tissue of the patients and found the mean of 2.87 ± 1.60 *μ*mol/mol DNA. To analyze the effects of AFB1 exposure on HCC prognosis, this variable was divided into two groups: low and high level. Results showed high AFB1 exposure levels were associated with decreasing 1-year, 3-year, and 5-year survival rate (Figure 1 and Table 2). Multivariate Cox regression analysis (with stepwise forward selection based on likelihood ratio test) exhibited that this variable increased dying risk and tumor reoccurring risk of HCC, with an HR of 2.12 and 2.40 (*P* < 0.01; Figure 1), respectively.

### 3.3. MiR-24 Was Upregulated in HCC Samples and in HCC Cells

**Figure 2:**
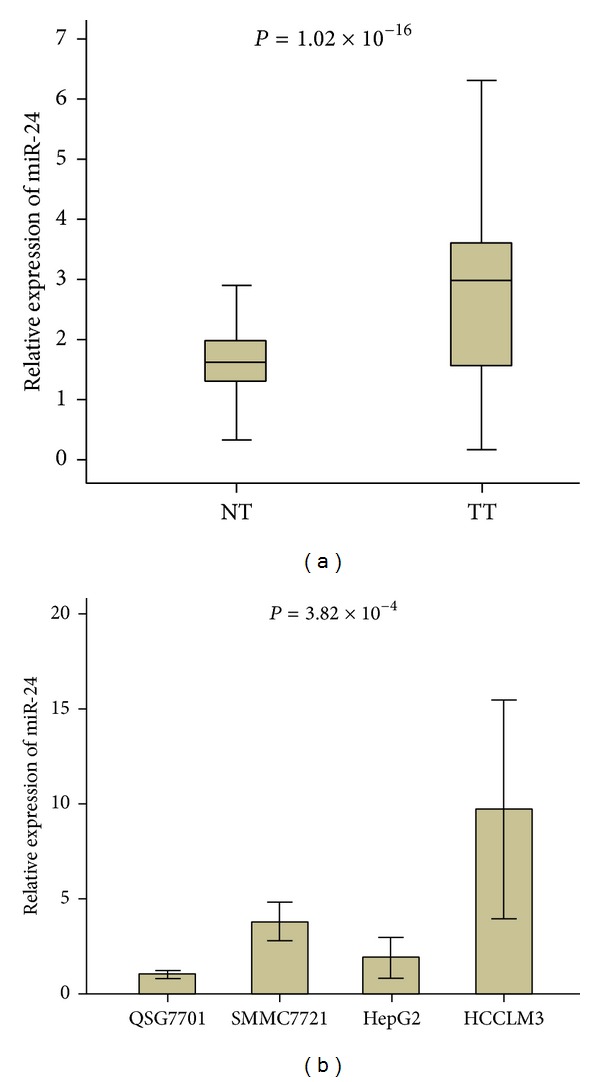
MiR-24 expression related with HCC tumorigenesis. (a) miR-24 expression was evaluated in the tumor tissues versus in the adjacent noncancerous tissues. The relative expression of miR-24 is shown as box plots, with horizontal lines representing the median, the bottom, and the top of the boxes representing the 25th and 75th percentiles, respectively, and vertical bars representing the range of data. We compared the difference among group using the Mann-Whitney *U* test. (b) miR-24 expression was higher in cancer cell line SMMC-7721 than in noncancer cell line QSG-7701.

We performed the real-time PCR experiment to detect the expression of mature miR-24 RNA in HCC tumor tissues and adjacent noncancerous tissues. Through comparing Ct values in these two types of tissues, we evaluated the significance of different miR-24 expression and observed that miR-24 expression was significantly higher in tumour tissues (TT) than in nonmalignant adjacent liver tissues (NT), with a relative expression value of 2.95 ± 1.88 versus 1.73 ± 0.92 (*P* < 0.01, Figure 2(a)). We also found similar results in the expression analysis of this microRNA in HCC cell lines and nontumor liver cell lines QSG7701 (*P* = 3.82 × 10^−4^, Figure 2(b)). Furthermore, higher expression of miR-24 was observed in the HCCLM3 (a kind of poor differentiated HCC cells with high infiltrating capacity) cells compared to in the HepG2 (a kind of good differentiated HCC cells with low infiltrating capacity).

### 3.4. MiR-24 Expression Was an Independent Factor of HCC Prognosis

**Figure 3:**
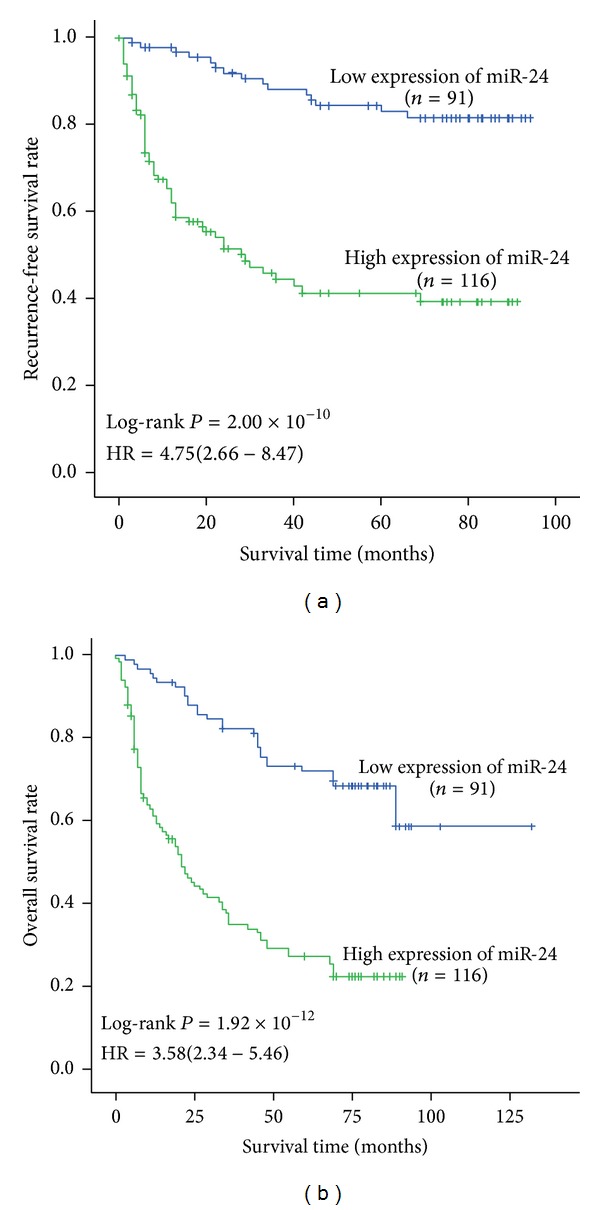
Association between survival and miR-24 expression in 207 HCC cases receiving curative treatment. According to the average expression in cancerous tissues, miR-24 expression was divided into two groups: low expression group (relative level ≤ 2) and high expression group (relative level > 2). MiR-24 expression was associated with tumor reoccurrence-free survival (a) and overall survival (b) of HCC. Cumulative hazard function was plotted by Kaplan-Meier's methodology, and *P* value was calculated with two-sided log-rank tests. Relative hazard ratio (HR) and corresponding 95% CI of high miR-24 expression (compared with low expression) was calculated using multivariable cox regression model (including all significant variables).

To investigate the effects of miR-24 expression on outcome of HCC patients, we divided miR-24 expression in cancerous tissues into two groups: low expression group (relative level ≤ 2.95) and high expression group (relative level > 2.95), according to the average relative expression levels. Kaplan-Meier survival analysis showed that patients with high miR-24 expression featured a significantly poorer prognosis than those with low miR-429 expression (*P* = 2.00 × 10^−10^ for RFS and* P* = 1.92 × 10^−12^ for OS, resp., Figure 3). Multivariate Cox regression analysis (with stepwise forward selection based on likelihood ratio test) was next performed to determine whether miR-24 expression was an independent predictor of HCC cases. The results exhibited that high miR-24 expression increased the risk of tumor reoccurrence compared with low expression (HR = 4.75, 95% CI = 2.66–8.47,* P* = 1.38 × 10^−7^, Figure 3(a)). Risk role was also found in the OS analysis; the corresponding HR (95% CI) was 3.58 (2.34–5.46), with a *P* value of 3.51 × 10^−9^ (Figure 3(b)). Taken together, these results suggested that this microRNA could be used as an independent prognostic marker for HCC.

### 3.5. Joint Effects of miR-24 Expression and AFB1 Exposure on HCC Prognosis

**Figure 4:**
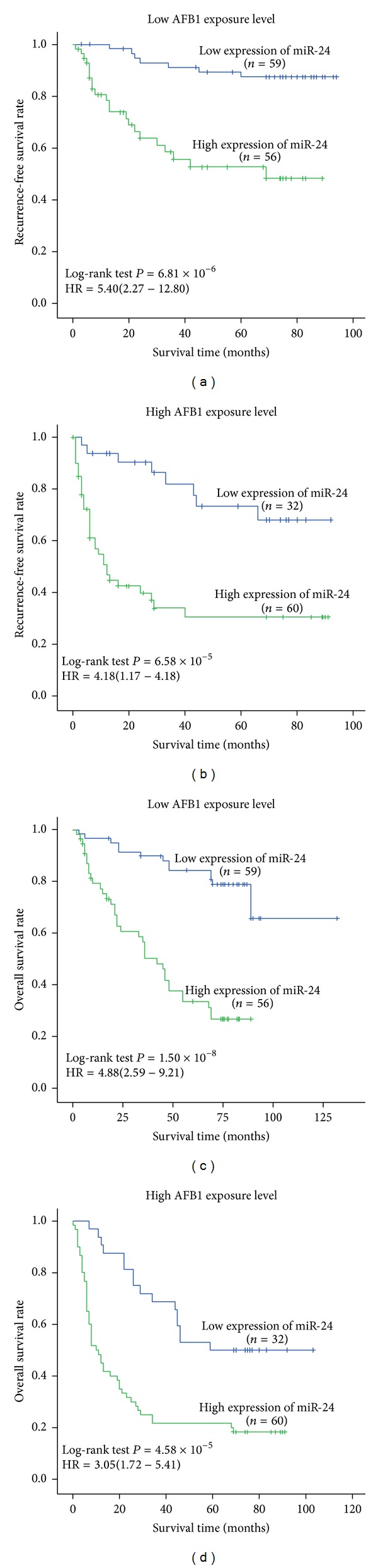
Survival analysis of miR-24 expression in strata of AFB1-DNA adduct. (a)-(b) Tumor recurrence-free survival (RFS) and miR-24 expression in strata of AFB1-DNA adduct levels. (c-d) Overall survival (OS) and miR-24 expression in strata of AFB1-DNA adduct levels. Cumulative hazard function was plotted by Kaplan-Meier's methodology, and *P* value was calculated with two-sided log-rank tests. Relative hazard ratio (HR) and corresponding 95% CI of high miR-24 expression (compared with low expression) was calculated using multivariable cox regression model (including all significant variables).

**Figure 5:**
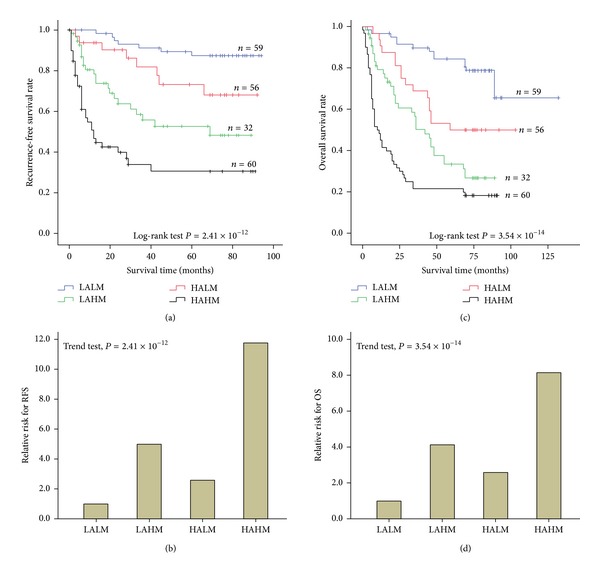
The joint effects of AFB1 exposure and miR-24 expression on HCC prognosis. (a and c) The joint effects of AFB1 exposure and miR-24 expression on tumor recurrence-free survival (RFS) and overall survival (OS) of HCC patients. Cumulative hazard function was plotted by Kaplan-Meier's methodology, and *P* value was calculated with two-sided log-rank tests. (b and d) Relative hazard ratio (HR) of both AFB1 exposure and miR-24 expression on HCC. HR was calculated using multivariable cox regression model (including all significant variables). Abbreviations: LALM, the combination of low AFB1 exposure level and low miR-24 expression; LAHM, the combination of low AFB1 exposure level and high miR-24 expression; HALM, the combination of high AFB1 exposure level and low miR-24 expression; HAHM, the combination of high AFB1 exposure level and high miR-24 expression.

To investigate the joint effects between miR-24 expression and AFB1 exposure on HCC prognosis, we performed a stratified analysis based on AFB1 exposure levels (Figure 4). We found lower 5-year RFS and OS for high miR-24 expression among these cases with high level of AFB1-DNA adducts (Figures 4(b) and 4(d)) than among those without high level of AFB1-DNA adducts (Figures 4(a) and 4(c)), suggesting high miR-24 expression might interact with AFB1 exposure in the process of HCC carcinogenesis. Next, a joint analysis between miR-24 expression and AFB1 exposure was accomplished (Figure 5). In this analysis, we used as a reference the lowest risk group: those who had both low AFB1-DNA adducts level and low miR-24 expression (LALM). Results showed that increasing adducts amount decreased 5-year survival rate of HCC; moreover, this effect was more pronounced among the high miR-24 expression subjects (Figures 5(a) and 5(c)). Moreover, compared to those with LALM, these with high AFB1-DNA adducts level and high miR-24 expression (HAHM) featured increasing tumor reoccurring risk (HR = 11.75, 95% CI = 5.15–26.79, Figure 5(b)) and death risk (HR = 8.13, 95% CI = 4.46–14.84, Figure 5(d)).

### 3.6. MiR-24 Expression Associated with the Clinic-Pathological Features of HCC Patients

To explore possible pathogenesis of miR-24 expression modifying the outcome of AFB1-related HCC, we analyzed the distribution difference of this microRNA expression among different clinic-pathological characteristics of cases. Results showed these HCC cases with high miR-24 expression, compared to those with low miR-24 expression, faced larger tumor size (OR = 2.01), lower tumor differentiation (OR = 2.10), and higher MVD (OR = 2.63, Table 3). However, the expression of this microRNA did not affect other features (data not shown).

### 3.7. MiR-24 Expression Modified HCC Cell Proliferation

**Figure 6:**
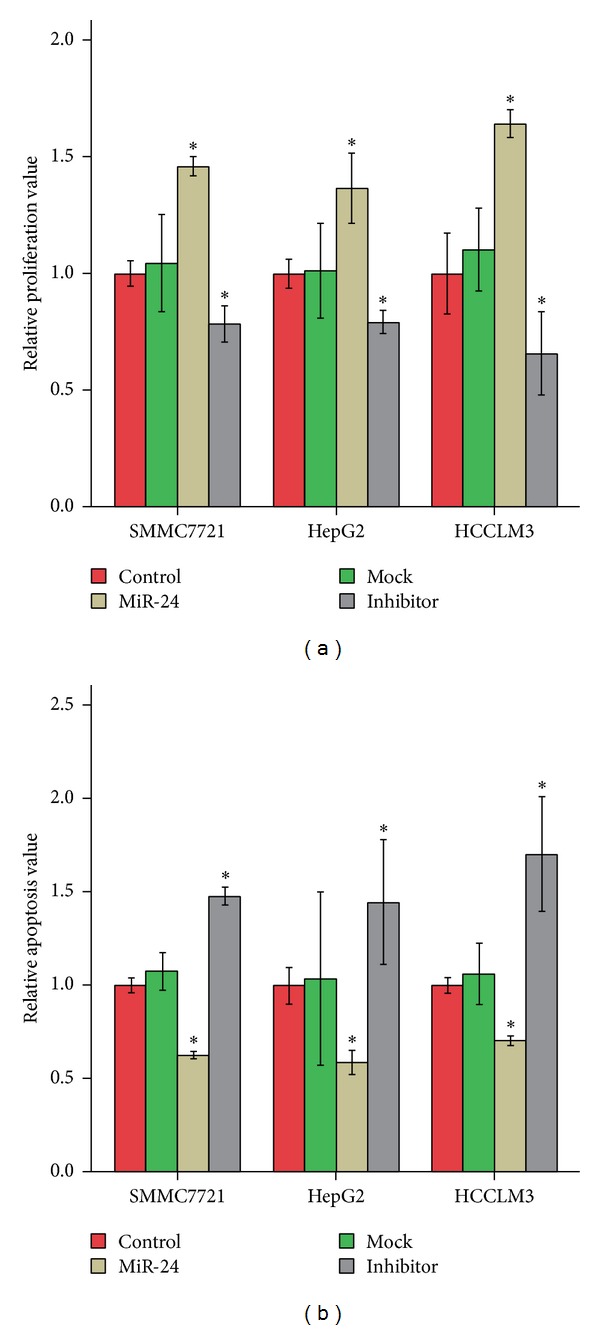
MiR-24 expression modified the proliferation and the apoptosis of HCC cancer cells. According to the types of mimics transfected, cell lines were divided into four groups: control group (control, by null mimics), mock group (mock, by mock mimics); miR-24 group (miR-24, by mature miR-24 mimics), and inhibitor group (inhibitor, by inhibitor of mature miR-24). (a) Association between miR-24 expression and cancer cell proliferation was elucidated using the CCK-8 assays. Relative proliferation value was calculated using control group as a reference. (b) Relationship between miR-24 expression and cancer cell apoptosis was evaluated by flow cytometry technique. Relative apoptosis value was calculated using control group as a reference. Data were analyzed using one-way ANOVA with Bonferroni corrections. Asterisk, *P* < 0.05.

We evaluated the functional role of miR-24 in HCC cells by means of measuring cell proliferation in HCC cells which were transfected with miR-24 mimics and its inhibitor. Overexpression of miR-24 in HCC cells promoted proliferation while downregulation of miR-24 in HCC cells inhibited cell proliferation. On the other hand, compared with the mock group, the proliferation of tumor cells in the inhibitor groups was inhibited significantly (*P* < 0.05, Figure 6(a)).

### 3.8. MiR-24 Expression Modulated the Apoptosis of HCC Cells

We also explored the function of miR-24 in HCC cells through analyzing changes in apoptosis after the HCC cells were transfected with miR-24 mimics and its inhibitor. DNA content of transiently microRNA-transfected cells was analyzed by flow cytometry. the RAV of SMMC7721 cell lines in the miR-24 group was significantly decreased (*P* < 0.05) compared with the control group. Tumor cell apoptosis in the inhibitor group, compared with the mock group, was promoted significantly (*P* < 0.05) (Figure 6(b)). Similar results were observed in the two other cells HepG2 and HCCLM3.

### 3.9. MiR-24 Expression Increased AFB1-DNA Adducts in the HCC Samples and SMMC-7721 Cells

**Figure 7:**
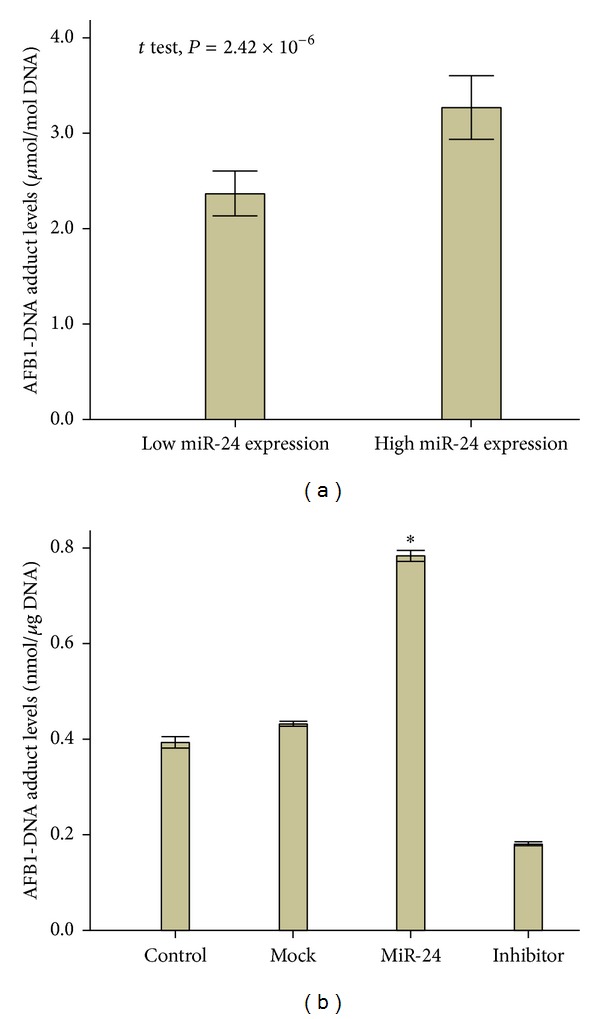
AFB1-DNA adducts formation in AFB1-treated SMMC7721 cells with overexpression of miR-24 (see Section 2). Levels of AFB1-DNA adducts were tested using comparative ELISA. Data were analyzed from three independent tests using one-way ANOVA with Bonferroni corrections. Asterisk, *P* < 0.05.

To investigate the effects of miR-24 expression on AFB1-DNA formation, we analyzed the effects of miR-24 expression on AFB1-DNA formation in liver cancer tissues. Results showed that these persons having high miR-24 expression in their tumor tissues faced increasing DNA adducts levels (3.27 ± 1.81 *μ*mol/mol DNA) compared with those with low miR-24 expression (2.37 ± 1.12 *μ*mol/mol DNA, *P* < 0.01, Figure 7(a)). A toxin experiment of AFB1 was next performed through transfecting different mimics into the SMMC7721 cells. We found that group with overexpression of miR-24 had elevated levels of AFB1-DNA adducts (0.795 ± 0.005 nmol/*μ*g DNA) compared with control group (0.394 ± 0.005 nmol/*μ*g DNA, *P* < 0.05, Figure 6(b)). On the other hand, compared with mock group (0.412 ± 0.002 nmol/*μ*g DNA), cells transfected by miR-24 inhibitor featured decreased levels of DNA adducts (0.181 ± 0.002 nmol/*μ*g DNA, *P* < 0.05, Figure 7(b)).

## 4. Discussion

In Guangxi Zhuang Autonomous Region, China, HCC is the most common cancer type, with an incidence rate of 53/100,000 per year and a death rate of 37–55/100,000 annually [1, 2]. Clinical-epidemiologic evidence suggests AFB1 exposure is a major risk factor for liver cancer in Guangxi Region [1]. AFB1 is an important I-type chemical carcinogen produced by some strains of the moulds aspergillus parasiticus and aspergillus flavus that grow readily on such foodstuffs as corn and groundnuts stored in damp conditions. Once ingested, this toxin is metabolized mainly by cytochrome P450 into the genotoxic metabolic aflatoxin B1-*exo*-8,9-epoxide (AFBO). AFBO is able to bind to DNA and causes genomic DNA damage and induces HCC [1, 2, 20]. In our study, about 2.9 *μ*mol/mol DNA of AFB1 adducts was tested in the liver cancer tissue samples, and AFB1-exposure status was also found to be associated with the poorer outcome of HCC. These results suggest that AFB1 is an important marker for HCC prognosis. However, because of metastasis or other causes, most AFB1-related HCC cases are already in an incurable stage with an extremely poor prognosis at the time of diagnosis [21]. Therefore, new prognosis biomarkers and therapies have been expected, but no remarkable advances have been made in the treatment and prognostic prediction of this malignant tumor.

Increasing evidence has shown that microRNAs may be a type of significant prognosis factor and potential therapeutic target for some malignant tumors including HCC [16, 22, 23]. MicroRNAs, a class of small noncoding single-stranded RNA with about 20 nucleotide sequence, are formed from the sequential processing of primary transcripts by two RNase enzymes, Drosha and Dicer [6, 24]. Through regulating gene expression, they functionally involve in cell differentiation, cell proliferation, cell apoptosis, physiological timing, metabolism, and hormone secretion [25, 26]. Moreover, increasing reports have exhibited that microRNAs may play a role in the aetiology and pathogenesis of various cancers by targeting a number of oncogenes or tumour suppressor genes [7]. The dysregulation of microRNA expression may correlate with the prognosis of some tumors such as HCC [16, 23].

Of particular recent interest is the possible contribution of miR-24 to tumor prognosis and tumorigenesis [23, 27, 28]. MiR-24, an important abundant microRNA encoded by the corresponding gene that maps to human chromosome 9q22 and 19p13 regions, is well conserved between various species and is expressed in normal tissues such as adipose tissue, mammary gland, kidney, and differentiated skeletal muscles [29]. Increasing evidence has shown that the miR-24 is frequently altered in liver cancer [11–13, 22, 23]. The dysregulation of miR-24 expression may modify tumor prognosis [22, 23]. In the present study, we collected 207 HCC tissue samples from Guangxi Zhuang Autonomous Region, both a high AFB1 exposure area and a high epidemic area of HCC in China, and investigated the possible effects of miR-24 expression on HCC prognosis. We found that HCC patients having high miR-24 expression in the tumor tissues had a significant poor RFS and OS compared with those with low expression of miR-24. Multivariate Cox regression analysis showed high miR-24 expression increased 3.75-times tumor reoccurrence risk and 2.58-times death risk; moreover, this risk did not depend on the clinic-pathological change. Supporting our results, a recent study has shown the dysregulation of miR-24 expression can modify the prognosis of cirrhotic HCC [22]. These data implied that miR-24 expression might be an independent prognostic factor for HCC and that its abnormal expression could be used as a prognostic marker for HCC.

In this study, we stratified HCC patients with respect to AFB1-DNA adducts levels and investigated the effects of miR-24 expression on HCC prognosis in different AFB1 exposure status. This was done primarily because the cause of HCC might modify the levels of miR-24 expression. Poorer outcome was found in these patients having high AFB1 exposure, suggesting possible interactive effects between environment exposure and gene expression on HCC prognosis. Next joint analysis proved this positive interaction. Therefore, it is well known that postoperative adjuvant therapy might significantly improve the prognosis of HCC cases, especially with high AFB1 exposure status.

Recent reports have exhibited that miR-24 may be involved in different cancers and play important role in carcinogenesis, such as gastric cancer [30], colorectal cancer [31], cervical cancer [32], oral squamous cell carcinoma [33], breast cancer [9], leukemia [34, 35], glioma [36], and lung cancer [10]. Several target genes of miR-24 have been discovered, including PKC-alpha, MXI 1, DHFR, ALK4, FAF1, DND1, AE1, p14ARF, and XIAP [9, 10, 27, 28, 30–42]. In this study, we also explored the association between miR-24 expression and HCC tumorigenesis through testing the expression difference of miR-24 in the different tissues (including tumor tissues and paired noncancerous matched tissues). Higher expression of miR-24 was observed in the tumor tissues, and this increasing expression of miR-24 was correlated with larger tumor size, tumor dedifferentiation, and increasing MVD. Our results also exhibited that the overexpression of miR-24 progressed cell proliferation and inhibited cell apoptosis. On the contrary, the suppression of miR-24 expression hindered cell proliferation and promoted cell apoptosis. Furthermore, different expression levels of miR-24 were found in the different degrees of differentiation; and HCC cells with poor differentiation and high infiltrating capacity had an increasing expression of miR-24. In accordance with our results, several recent studies have demonstrated that upregulation of miR-24 is involved in the tumorigenesis of HCC [11–13, 23, 43, 44]. Taken together, these results suggested that the dysregulation of miR-24 expression might play an important role in the tumorigenesis of HCC through promoting tumor angiogenesis, proliferation, tumor invasion, and metastasis [11–13].

Interestingly, we found that high expression of miR-24 could promote AFB1-DNA formation and increase adducts mount. This is possibly because miR-24 can target some detoxification enzyme genes [45] and reduce their detoxification capacity and subsequently result in the accumulation of AFB1-DNA adducts. Additionally, our previous reports showed that low expression of DNA repair by microRNA would decrease the DNA repair capacity and subsequently increase DNA damage amount and HCC risk [14–16]. These results provided new insights into the mechanism of HCC induced by AFB1.

The present study had several limitations. Only 207 HCC patients were enrolled in the analysis of the clinic-pathological characteristics and prognosis. We would like to confirm the findings in a larger HCC patient population. Another important limitation was that we did not do migration and invasiveness assays to validate the involvement of miR-24 in tumour migration and invasion. Although the status of miR-24 expression was investigated in cases of HCC, other microRNAs, such as microRNA-629 and microRNA-124, which may be involved in HCC tumorigenesis and modify HCC prognosis [11], were not evaluated. Additionally, because the liver disease itself may affect the metabolism of AFB1 and modify the levels of AFB1 DNA adducts, the increased death risk and tumor reoccurring risk with AFB1 exposure status noted in this study was probably underestimated. Therefore, more microRNAs deserve further elucidation based on a large sample and the combination of genes and AFB1 exposure.

## 5. Conclusions

In summary, this study is, to the best of our knowledge, the first report that describes miR-24 expression in AFB1-related liver cancer and its associations with HCC prognosis. Our results showed that miR-24, as an oncogene, was overexpressed in liver cancer tissues and could be considered as a potential prognostic factor for HCC. Furthermore, overexpression of this microRNA was associated with AFB1-related HCC tumorigenesis. Therefore, more detailed molecular pathogenesis analysis deserves elucidation based on the results from large samples. Expanding insights into the key role of dysregulated microRNAs involved in liver tumorigenesis will yield important clues for the complicated molecular pathogenesis of HCC and may assist in the development of new therapeutic regimens for HCC patients, especially from high AFB1 exposure areas.

## Figures and Tables

**Table 1:** Characteristics of the patients with HCC.

Characteristics	
Age, year	
Mean ± SE	46.9 ± 11.5
Range	15–75
Sex	
Man, *n* (%)	189 (91.3)
Female, *n* (%)	18 (8.7)
Ethnicity	
Han, *n* (%)	163 (78.7)
Minority, *n* (%)	44 (21.3)
HBV infection	
HBsAg (−), *n* (%)	33 (15.9)
HBsAg (+), *n* (%)	174 (84.1)
HCV infection	
anti-HCV (−), *n* (%)	204 (98.6)
anti-HCV (+), *n* (%)	3 (1.4)
Smoking status	
No, *n* (%)	165 (79.7)
Yes, *n* (%)	42 (20.3)
Drinking status	
No, *n* (%)	167 (80.7)
Yes, *n* (%)	40 (19.3)
Liver cirrhosis	
No, *n* (%)	41 (19.8)
Yes, *n* (%)	166 (80.2)
TNM stage	
I, *n* (%)	13 (6.3)
II, *n* (%)	194 (93.7)
Tumor size	
≤5 cm, *n* (%)	101 (48.8)
>5 cm, *n* (%)	106 (51.2)
Tumor grade	
I	44 (21.3)
II	106 (51.2)
III	54 (26.1)
IV	3 (1.4)

Total, *n* (%)	207 (100)

**Table 2:** RFS rate and OS rate of HCC cases.

		RFS rate (%)			OS rate (%)		
		1 year	3 years	5 years	1 year	3 years	5 years
Figure 1	AFB1-DNA adduct level						
	Low	89.9	75.5	70.8	88.4	74.4	60.9
	High	66.7	53.9	46.7	64.1	41.3	31.5

Figure 3	miR-24 expression						
	Low	97.8	88.1	82.9	94.5	82.3	72.0
	High	65.2	44.4	41.2	61.1	37.7	27.3

Figures 4(a) and 4(c)	miR-24 expression						
	Low	100	91.1	87.3	96.6	89.7	84.3
	High	86.8	55.7	52.6	79.2	56.5	33.5

Figures 4(b) and 4(d)	miR-24 expression						
	Low	93.8	81.9	73.2	90.6	68.8	50.0
	High	50.7	33.9	30.5	48.3	25.0	20.0

**Table 3:** Expression levels of miR-24 and clinicopathological features of cases.

	Low expression		High expression		OR (95% CI)^a^	*P *
	*n *	%	*n *	%		
AFB1 exposure						3.10 × 10^−2^
Low level	59	64.8	56	48.3	Reference	
High level	32	35.2	60	51.7	1.92 (1.06–3.46)	
Tumor size						1.91 × 10^−2^
≤5 cm	53	58.2	48	41.4	Reference	
>5 cm	38	41.8	68	58.6	2.01 (1.12–3.60)	
Tumor grade						3.92 × 10^−2^
I-II	72	79.1	78	67.3	Reference	
III-IV	19	20.9	38	32.7	2.10 (1.04–4.27)	
MVD						1.93 × 10^−2^
Low	50	54.9	38	32.8	Reference	
High	41	45.1	78	67.2	2.62 (1.43–4.81)	

^a^Adjusted by age, sex, race, HBV and HCV infection status, and smoking and drinking status.
